# Nanosized titanium dioxide particle emission potential from a commercial indoor air purifier photocatalytic surface: A case study

**DOI:** 10.12688/openreseurope.14771.1

**Published:** 2022-07-04

**Authors:** Antti Joonas Koivisto, Sara Trabucco, Fabrizio Ravegnani, Francescopiero Calzolari, Alessia Nicosia, Benedetta Del Secco, Marko Altin, Elisa Morabito, Magda Blosi, Anna Costa, Franco Belosi

**Affiliations:** 1 Air Pollution Management (APM), Mattilanmäki 38, 33610 Tampere, Finland; 2 Institute for Atmospheric and Earth System Research (INAR), University of Helsinki, PL 64, FI-00014, Helsinki, Finland; 3 ARCHE Consulting, Liefkensstraat 35D, B-9032 Wondelgem, Belgium; 4 ISAC-CRN, Institute of Atmospheric Sciences and Climate, National Research Council of Italy, Via Gobetti, 101, 40129 Bologna, Italy; 5 Witek srl, Via Siena 47, 50142 Firenze, 50142, Italy; 6 Department of Environmental Sciences, Informatics and Statistics, Cá Foscari University, Via Torino 155, 30172 Venice, Italy; 7 ISTEC-CNR, Institute of Science and Technology for Ceramics, National Research Council, Via Granarolo 64, 48018 Faenza, Italy

**Keywords:** Nanomaterial, TiO2, indoor air purifier, photocatalytic oxidation, release, emission, exposure, indoor aerosol

## Abstract

**Background:** Photocatalytic air purifiers based on nano-titanium dioxide (TiO _2_) visible light activation provide an efficient solution for removing and degrading contaminants in air. The potential detachment of TiO _2_ particles from the air purifier to indoor air could cause a safety concern. A TiO _2_ release potential was measured for one commercially available photocatalytic air purifier “Gearbox Wivactive” to ensure a successful implementation of the photocatalytic air purifying technology.

**Methods:** In this study, the TiO _2_ release was studied under laboratory-simulated conditions from a  Gearbox Wivactive consisting of ceramic honeycombs coated with photocatalytic nitrogen doped TiO _2_ particles. The TiO _2_ particle release factor was measured in scalable units according to the photoactive surface area and volume flow (TiO _2_-ng/m ^2^×m ^3^). The impact of  Gearbox Wivactive on indoor concentration level under reasonable worst-case conditions was predicted by using the release factor and a well-mixed indoor aerosol model.

**Results:** The instrumentation and experimental setup was not sufficiently sensitive to quantify the emissions from the photoactive surfaces. The upper limit for TiO _2_ mass release was <185×10 ^-3^ TiO _2_-ng/m ^2^×m ^3^. Under realistic conditions the TiO _2_ concentration level in a 20 m ^3^ room ventilated at rate of 0.5 1/h and containing two Gearbox Wivactive units resulted <20×10 ^-3^ TiO _2_-ng/m ^3^.

**Conclusions:** The release potential was quantified for a photocatalytic surface in generalized units that can be used to calculate the emission potential for different photocatalytic surfaces used in various operational conditions. This study shows that the TiO _2_ nanoparticle release potential was low in this case and the release does not cause relevant exposure as compared to proposed occupational exposure limit values for nanosized TiO _2_. The TiO _2_ release risk was adequately controlled under reasonable worst-case operational conditions.

## Plain language summary

Photocatalytic oxidation is a widely used technique to degrade air pollutants into non-toxic or less harmful forms and to deactivate viruses and bacteria. Visible light can activate nitrogen doped nanosized titanium dioxide (TiO _2_N) that makes it a promising material in air purification devices. The potential release of nanosized TiO _2_ is one risk factor that should be addressed adequately when applying this novel technology. Here we studied a release of TiO _2_ nanoparticles from a commercially available air purifier called Gearbox Wivactive. The release factor was calculated in a generalized unit that can be used to calculate the emission potential for different photocatalytic surfaces used in various operational conditions. In this case study, it was possible to measure only upper estimate (<48×10 ^-3^ TiO _2_-ng/m ^2^×m ^3^) for the Gearbox Wivactive photocatalytic surfaces. The risk related to the TiO _2_ release potential was considered adequately controlled because the TiO _2_ concentration level under reasonable worst-case conditions was below the proposed occupational exposure limit values for nanosized TiO _2_.

## List of abbreviations

**Table:** 

ASINA	Anticipating Safety Issues at the Design Stage of NAno Product Development
CPC	Condensation particle counter
ICP-MS	Inductively coupled plasma mass spectrometry
iCAP RQ	Inductively-coupled argon-plasma radio quadrupole
OEL	Occupational exposure limit
OPC-N3	Optical particle counter Alphasense
PM	Particulate matter
REACH	Registration, Evaluation, Authorisation and Restriction of Chemicals
TiO _2_	Titanium dioxide
TiO _2_N	Nitrogen doped titanium dioxide

## Introduction

Photocatalytic oxidation is the most promising technology for air purification. It can degrade diverse air pollutants into non-toxic or less harmful forms and deactivate viruses and bacteria using visible light under ambient conditions ( Ângelo *et al*., 2013; Chen *et al*., 2015; Kotzias *et al*., 2022; Poorhosseini *et al*., 2011; Weon *et al*., 2019). Nanosized titanium dioxide (TiO _2_) and its modifications is the most adopted photocatalytic material for air treatment devices holding over 60% of the patents published between 2010 and 2020 ( Muscetta & Russo, 2021). Current research and development is mainly focused on TiO _2_ photocatalysis ( He *et al*., 2021). However, there is a concern on their safety under real indoor conditions which might limit their diffusion. Among the main challenges, these air purifiers should address there are the control of the pollutant degradation mechanism mineralization with drawbacks due to by-product formation ( Vasilache *et al*., 2013) and the adhesion of the photocatalyst to the substrate with potential drawbacks due to nanoparticles detachment. According to the authors knowledge, the potential release of nanoparticles (NPs) from air purifiers has not been studied previously (see also Koivisto *et al*., 2017).

This study investigates the release of NPs from the photocatalytic air purifier “Gearbox Wivactive” manufactured by WITEK srl. The Gearbox Wivactive was selected as WITEK srl is part of the Anticipating Safety Issues at the Design Stage of NAno Product Development (ASINA) project with patented light technology for air purification by nano-titanium. The air purifier Gearbox Wivactive contains ceramic honeycombs coated with nanosized TiO _2_N, which is widely used in photocatalytic oxidation activated by visible light ( Natarajan *et al*., 2021). The TiO _2_ is a semiconductor with an energy gap equal to *E _g_* = 3.2 eV, if it is irradiated with photons of energy greater than *E _g_* (wavelength less than 388 nm), an electron is able to overcome the energy gap and is promoted from the valence band to the conduction band, while in the TiO _2_N the energy gap is *E _g_* = 2.7eV-2.9eV. Therefore, TiO _2_N is able to get significantly excited also at visible wavelengths.

The activity was carried out in the framework of the European Union’s Horizon 2020 ASINA project Task 2.1 "Identifying and quantifying release during all stages of nano-enabled products life-cycle" (see *Grant information*). The NP emissions were characterized for the Gearbox Wivactive as TiO _2_-ng/(honeycomb × m ^3^ of ventilated air) and in generic form as TiO _2_-ng/(m ^2^ of photoactive surface area × m ^3^ of ventilated air). The release rates were used to predict the TiO _2_ exposure potential under relevant indoor conditions. The TiO _2_N exposure risk was estimated by using a proposed occupational exposure limit (OEL) value for nanosized TiO _2_.

## Methods

Nanoparticle release tests were performed by using the modified air purifier under laboratory-simulated conditions. The exposure potential by nanoparticle release was estimated by using a single compartment mass flow model.

### Gearbox Wivactive air purifier configuration

**Figure 1.**
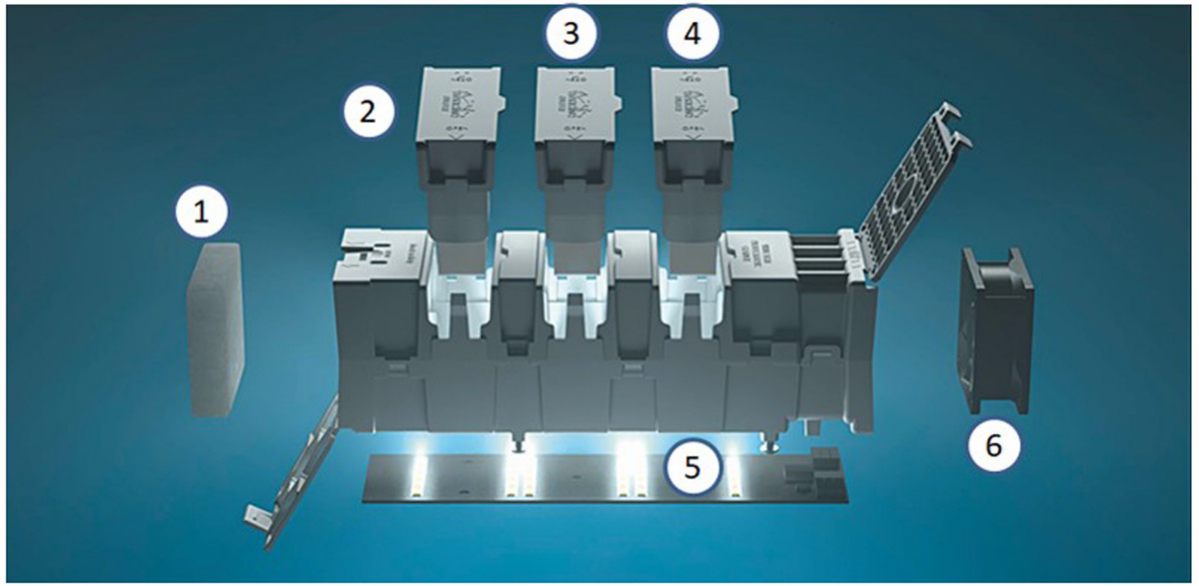
Gearbox Wivactive air purifier main components (reproduced with permission from the copyright holder, WITEK ( WITEK, 2022)). 1) a prefilter, 2,3 and 4) photoactive honeycombs, 5) a light emitting diode light, 6) a fan.

The working principle of the air purifier is shown in Figure 1. The system consists of a class G4 ( EN 779:2012) prefilter (1) that removes the coarse fraction (particle diameter *ca.* >10 μm) of the airborne particles and microorganisms), three ceramic honeycombs coated with TiO _2_N (2,3,4) and led light system (5) that activate the release of free oxidant radicals and a fan maintaining a flow through the system (6). The honeycombs are coated with TiO _2_N suspension prepared by Colorobbia Italia, SPA (patent no. EP3788009A1; Sovigliana Nanomaterials Vinci, FI, Italy). Single honeycomb inner surface area ranges from 90 to 100 cm ^2^.

Two different experimental set-ups were considered by assembling specific Gearbox Wivactive units:

- Test 1: Close, static system by assembling a test unit from components 2–6 (Gearbox Wivactive modified A)
- Test 2: Open, dynamic system, by assembling a test unit from components 2–5 (Gearbox Wivactive modified B).

### Instrumentation

Particle number concentrations were measured from 4 nm by using a Condensation Particle Counter (CPC, TSI mod. 3775) at sample flow 5 cm ^3^/s and from *ca.* 300 nm by using an optical particle counter Alphasense (OPC-N3). OFF-line gravimetric particulate matter (PM) samples (total fraction) were taken by collecting particles on an absolute quartz fiber filter (Sartorius, grade T293, Goettingen, Germany). The filters were analyzed by using inductively-coupled argon-plasma radio quadrupole (iCAP RQ) mass spectrometer (ICP-MS; Thermo Fisher Scientific, Environmental Department of Cà Foscari University, Venice; lower limit of quantification (LOQ) is 1 ng). The flow rate was determined by averaging the inlet velocities measured by means of a hot wire anemometer (Terman ANM-0/B, LSI spa, Milan, Italy).

### Experimental setup in Test 1

**Figure 2.**
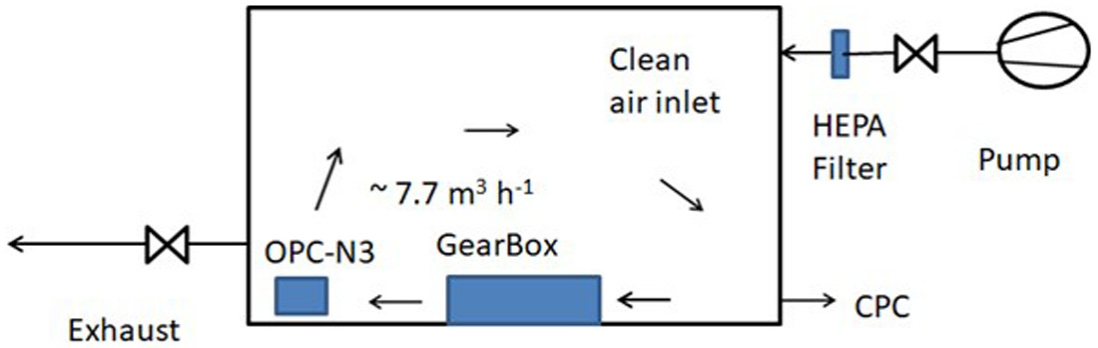
Test 1 experimental set up where the Gearbox Wivactive recirculates the air inside a closed chamber. CPC, condensation particle counter; OPC-N3, optical particle counter Alphasense; HEPA, high efficiency particulate air.

Test 1 was performed in a closed chamber with a volume of 220 L ( Figure 2). Before measurements, the chamber was ventilated with filtered laboratory air (Quartz-Microfibers filter- Grade T293, Sartorius). The modified Gearbox Wivactive A was operated by using the standard fan (6) at flow rate of 7.7 m ^3^/h. The flow rate was determined by averaging the inlet velocities measured by means of a hot wire anemometer and considering a 7 cm × 6.6 cm inlet surface. This corresponds to about 30 air changes per hour.

The chamber total particle number concentration was measured continuously with the OPC-N3 recirculating the air inside the chamber. An external CPC was used to measure particle number concentration for *ca.* 1-minute at morning, noon and afternoon. This minimizes particle flow from laboratory air to the chamber. Tests were carried out continuously for three days which corresponds to *ca.* 540 m ^3^ recirculated air volume.

Assuming fully mixed concentrations inside the chamber the mass balance of particles is


$$V\frac{dC(t)}{dt}=G+C_{room}(t)\cdot P\cdot Q-(\lambda \cdot V-Q)C(t)\;(1)$$

Where *V* (m ^3^) is the chamber volume, *C*( *t*) (1/m ^3^) is the number concentration of contaminants in the chamber, *t* is the time (s), *G* (1/s) is the release rate of airborne particles, *C _room_* ( *t*) (1/m ^3^) is the laboratory room air concentration which penetrates the chamber at efficiency of *P* (-), *Q* (m ^3^/s) is the chamber ventilation air volume flow rate, and *λ* (1/s) is the particle loss rate onto the surfaces inside the chamber. When assuming *Q* ≈ 0 m ^3^/s and particle losses on the surfaces *λ* ≈ 0 1/s the mass balance equation is simplified to


$$V\frac{dC(t)}{dt}=G\;(2)$$

This can be used to estimate the emission rate from the OPC-N3 concentration time series.

### Experimental setup in Test 2

**Figure 3.**
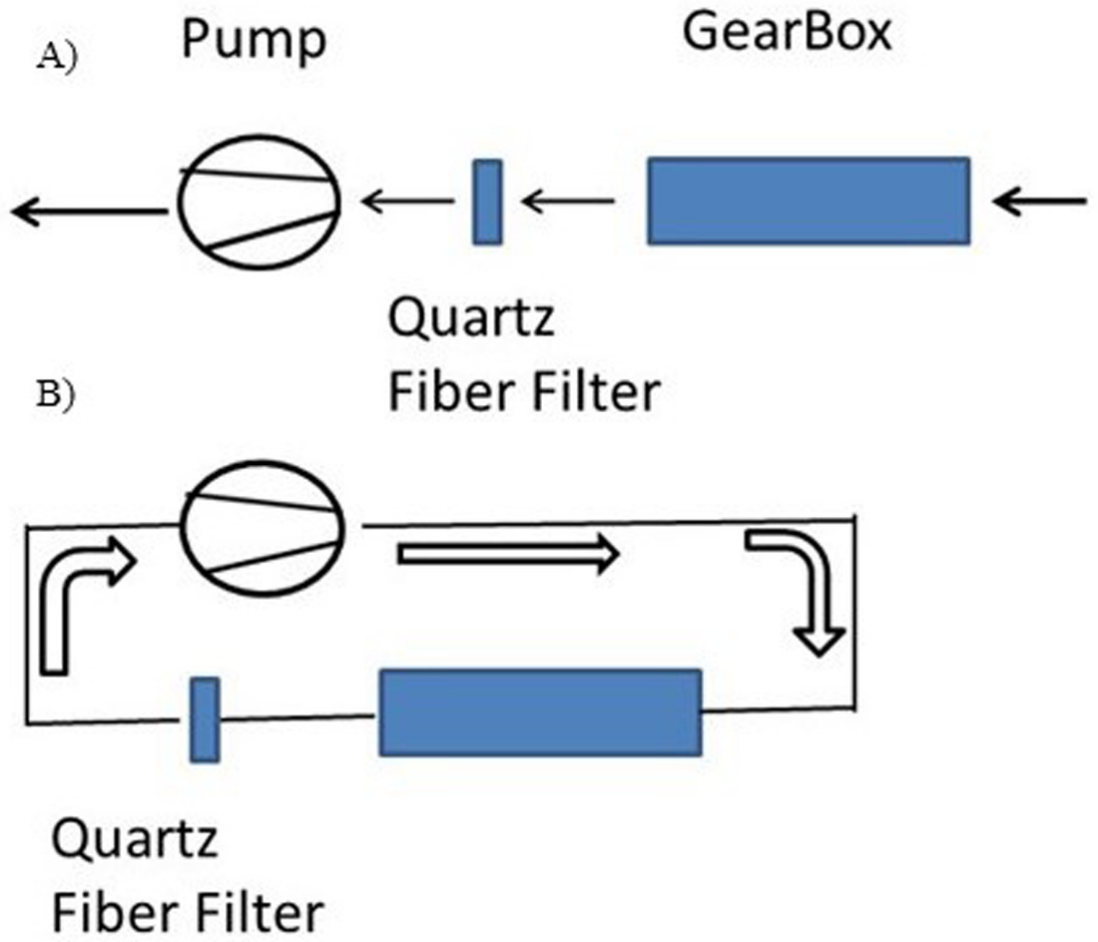
Scheme of the Test 2 experimental set up where **A**) laboratory air is aspirated through the Gearbox Wivactive and **B**) air is recirculated in a sealed circuit.

Test 2 was carried out by using the modified Gearbox Wivactive B connected to the quartz filter and maintaining 4.4 m ^3^/h air flow by using an external pump (Bravo H-Plus, TCR Tecora, Cogliate, Italy). Two different configurations were used: Test 2A was performed with laboratory air and Test 2B was performed by using recirculated air ( Figure 3). Particle release was estimated by collecting Ti mass on a quartz filter and calculated as mass of TiO _2_ per ventilation volume (TiO _2_-ng/m ^3^). In Test 2A it is assumed that the laboratory room air does not contain Ti and the emission rate is calculated by assuming that Ti release is independent of the Gearbox Wivactive volume flow rate.

### Exposure potential simulation

The reasonable worst-case simulation was performed by using a single compartment model where air is considered to be fully mixed all the time. The Gearbox Wivactive is assumed to operate without G4 prefilter (component 1 in Figure 1) and the TiO _2_ particle filtration is considered insignificant. The Gearbox Wivactive flow rate is assumed to be 20 m ^3^/h and two Gearbox Wivactive systems are assumed to operating in a 20 m ^3^ room. This corresponds to 2 1/h air recirculation rate through the two air purifiers. The room general ventilation is assumed to be 0.5 1/h representing typical ventilation rate in European dwellings ( Dimitroulopoulou, 2012).

Modelling was performed using a deterministic single compartment model (Task Exposure Assessment Simulator ( TEAS) 2019 V 1.00, model No. 101, Exposure Assessment Solutions, Inc., Morgantown, MI, USA). TEAS is a commercial software. The model is described in detail by Hewett & Ganser (2017). A free, alternative model is IH Mod 2.0 (requires Microsoft Excel).

## Results

### Emission measurements

In Test 1, CPC measurements increased the particle number concentrations during the one-minute measurement period due to external air infiltration to replace the sampled volume. For further information, see *Underlying data* ( Koivisto *et al*., 2022).

**Figure 4.**
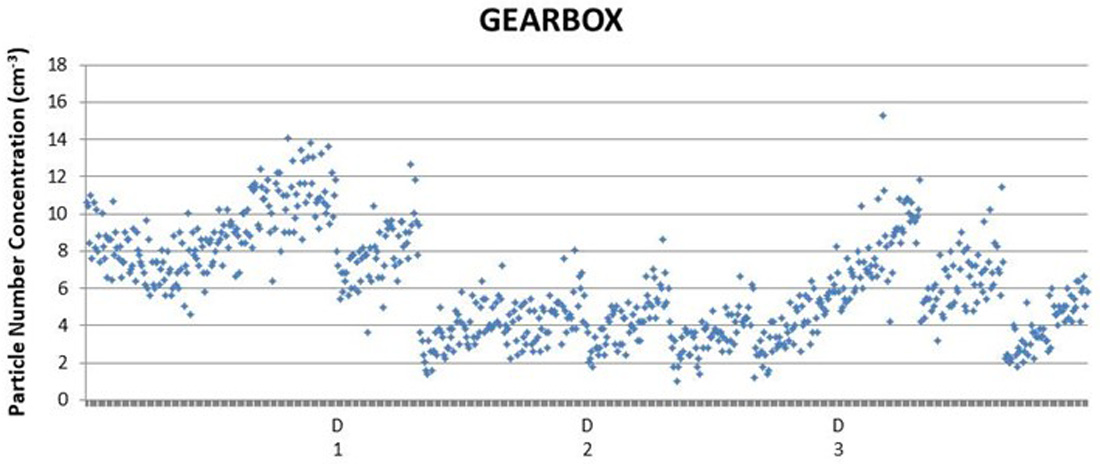
Single condensation particle counter measurements over three days. The concentration pattern is increasing due to external air infiltration ( Koivisto *et al*., 2022).

The background concentration varied from *ca.* 4 to 14 1/cm ^3^ prior switching the Gearbox Wivactive on after which the concentrations varied from *ca.* 1 to 16 1/cm ^3^ ( Figure 4).

**Figure 5.**
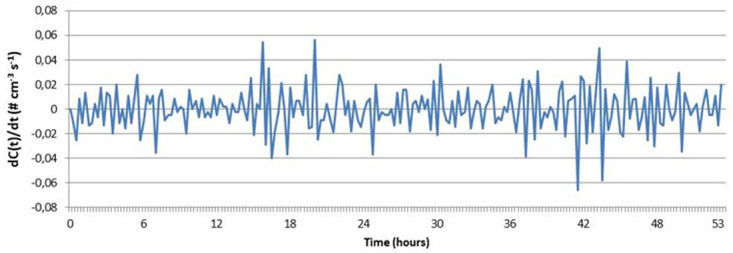
Time derivative of the particle number concentration inside the box (each point is 15 minutes averaged concentration). See Koivisto *et al*., 2022.

The particle number concentration time derivative was calculated for the OPC-N3 measurement when CPC sampling was not performed. The average emission rate was 1×10 ^-4^ ± 0.02 1/(cm ^3^×s) where variation is one standard deviation ( Figure 5). Assuming fully mixed concentrations and no particle losses inside the chamber, the emission rate was up to 4400 1/s (when given as one standard deviation upper range) according to the OPC-N2. The result is not considered as reliable because of the high lower particle count limit in OPC-N2 and large chamber volume of 220 L. The result can be used as indicative evidence for low particle release.

Particles collected in Test 2A and B to quartz fiber filter samples and the blank field filters sampling from laboratory air were analyzed by means of the iCAP RQ ICP-MS; see *Underlying data* ( Koivisto *et al*., 2022). The amount of Ti was below the lower LOQ in all samples. Table 1 shows the sampling parameters and the upper estimates for the release as Ti and TiO _2_.

**Table 1.** Test 2A and 2B Ti (titanium) emission measurements given as release per ventilation volume (ng/m ^3^) and emission rate (ng/h) for a single Gearbox Wivactive system containing three photoactive honeycombs units and in generalized units as release per surface area and ventilation volume. For more information see *Underlying data* ( Koivisto *et al*., 2022). TiO _2_ emissions were calculated from Ti gravimetric mass and atomic masses of Ti and TiO _2_ ( Koivisto *et al*., 2022).

Test	Sampling time (h)	Volume (m ^3^)	Ti (ng/m ^3^)	TiO _2_ (ng/m ^3^)	TiO _2_ (ng/h)	TiO _2_ (ng/m ^2^×m ^3^)
Test 2A	96	303.7	< 3×10 ^-3^	< 5×10 ^-3^	<1.7×10 ^-2^	<185×10 ^-3^
Test 2B	72	229.6	< 4×10 ^-3^	< 7×10 ^-3^	<2.2×10 ^-2^	<259×10 ^-3^

### Exposure potential simulation

The upper limit for TiO _2_ mass emission measured in Test 2A was used to predict the maximum exposure potential by using the TEAS model no. 101. The emission rate for different volume flows and coated surface areas can be calculated by using Test 2A release factor of <185×10 ^-3^ TiO _2_-ng/m ^2^×m ^3^. Assuming that two Gearbox Wivactive systems are operated at 10 m ^3^/h each the emission rate would be <3.7 TiO _2_-ng/min for a total photoactive surface area 0.06 m ^2^ and a flow rate 0.33 m ^3^/min. In a 20 m ^3^ ventilated at 0.5 1/h rate and assuming the air fully mixed, the concentration after 10 h continuous run reaches steady-state level at <20×10 ^-3^ TiO _2_-ng/m ^3^; see Koivisto *et al*. (2022).

## Discussion

The release was not possible to quantify within these experiments due to low release as compared to the instruments lower LOQ. The TiO _2_ mass release was <185×10 ^-3^ TiO _2_-ng/m ^2^×m ^3^. Under realistic conditions the TiO _2_ concentration level would increase to <20×10 ^-3^ TiO _2_-ng/m ^3^. Current proposed OELs vary for nano-TiO _2_ from 0.8 to 5000 μg/m ^3^ when given in different size fractions and specified under different experimental conditions for 8-h time weighted average ( ANSES, 2021; Mihalache *et al*., 2017; WHO, 2017). For 24-h exposure the limit would be three times lower that would mimic public population exposure. Because there are no legally binging limit values for nanosized TiO _2_ it is not possible perform regulatory risk assessment under Registration, Evaluation, Authorisation and Restriction of Chemicals (REACH) framework ( ECHA, 2016a; ECHA, 2016b). However, the indicative analysis shows that the Gearbox Wivactive does not release TiO _2_ nanoparticles in relevant quantities as compared to the current proposed occupational exposure limit values for nanosized TiO _2_. Release factor given in general form can be used to estimate the upper limit of release for different photocatalytic surface and volume flow settings.

## Conclusions

The potential release of particles from the air purifier Gearbox Wivactive (Ti-coated honeycombs units) has been investigated by means of a CPC (particles above 4 nm) and an OPC-N3 (particles above 300 nm) in a close chamber and total Ti-mass collected on a quartz filter in an open and sealed-circuit system. Chamber studies did not have a sufficient sensitivity to detect low particle concentrations in below few 1/cm ^3^ to quantify emissions at sufficient precision. However, indicative emission of 4400 1/s quantified from OPC-N3 measurements shows low emissions. Chemical analysis of quartz filter samples revealed that the release is below <185×10 ^-3^ TiO _2_-ng/m ^2^×m ^3^. The release factor can be used to predict emissions from different TiO _2_N based photocatalytic surfaces. Under reasonable indoor conditions, the maximum exposure potential was <20×10 ^-3^ TiO _2_-ng/m ^3^ when using two Gearbox Wivactive air purifiers over 10 h. Preliminary risk assessment based on proposed OELs shows adequately controlled risk related to TiO _2_ release potential.

## Data availability

### Underlying data

Zenodo: Nanosized titanium dioxide particle emission potential from a commercial indoor air purifier photocatalytic surface – A case study. https://doi.org/10.5281/zenodo.6547915 ( Koivisto *et al*., 2022).

This project contains the following underlying data:

- CPC.xlsx (Condensation particle counter measurement file).
- OPC Alphasense.xlsx (OPC Alphasense measurement file).
- ICP-Mass.xlsx (ICP mass measurement file).
- GearBox TEAS simulation.pdf (Exposure potential simulation file generated by TEAS).
- Modeling.xlsx (Calculation of the simulation parameters).

Data are available under the terms of the Creative Commons Attribution 4.0 International license (CC-BY 4.0).

## Ethics and consent

Ethical approval and consent were not required.
