# Supplementary material for: Nanosized titanium dioxide particle emission potential from a commercial indoor air purifier photocatalytic surface: A case study
Source: Open Res Eur. 2022 Jul 4;2:84. [Version 1] doi: 10.12688/openreseurope.14771.1 (PMC10446146; doi:10.12688/openreseurope.14771.1)
Supplement: Peer review history

# Peer review: Reviewer response for version 1

**DOI:** 10.21956/openreseurope.15956.r32164

**Published:** 2023-07-13

**Authors:** Julie Hot

**Affiliations:** 1 LMDC, INSA/UPS Génie Civil, Toulouse, France

This paper deals with the TiO2 release for a commercial photocatalytic air purifier “Gearbox Wivactive”. The experiments were carried out at a laboratory scale. The study is interesting because it focuses on a challenging issue associated with photocatalytic products, namely the release of nanoparticles.

 Here are few remarks:

- Introduction: Remove the comma after the word challenges -> “Among the main challenges these…”, remove the “s” after “authors” -> “according to the author knowledge” or write “authors’ knowledge”
- Introduction: You mentioned that NP emissions are expressed as “TiO2-ng/(honeycomb × m3 of ventilated air) and in generic form as TiO2-ng/(m2 of photoactive surface area × m3 of ventilated air)”. So, when you expressed quantitate results throughout the article, you should write ng/(m2xm3) (with brackets) and not ng/m2xm3. Moreover, is m2 not missing before “honeycomb”?
- Introduction: The following sentence is too long: “The TiO2 is a semiconductor with an energy gap equal to Eg = 3.2 eV, if it is irradiated with photons of energy greater than Eg (wavelength less than 388 nm), an electron is able to overcome the energy gap and is promoted from the valence band to the conduction band, while in the TiO2N the energy gap is Eg = 2.7eV-2.9eV.”
- Methods/Gearbox Wivactive air purifier configuration: Remove “)” after microorganisms.
- Methods/Gearbox Wivactive air purifier configuration: What was the purpose of running two different tests? Please explain your choice. Moreover, Test 1 was described as “static” but was carried out with a fan. It is confusing.
- Methods/Experimental setup in Test 2: Why were the two configurations Test 2a and 2B used?
- Results/Emission measurements: OPC-N2 was mentioned. OPC-N3 should be written instead.
- Results/Exposure potential simulation: Please check the result 3.7 TiO2-ng/min.
- Discussion: Add “to” -> “it is not possible to perform”.

I agree to the passing peer review of this article after these minor modifications.

Is the study design appropriate and does the work have academic merit?

Yes

Is the work clearly and accurately presented and does it cite the current literature?

Yes

If applicable, is the statistical analysis and its interpretation appropriate?

Yes

Are all the source data underlying the results available to ensure full reproducibility?

Partly

Are the conclusions drawn adequately supported by the results?

Yes

Are sufficient details of methods and analysis provided to allow replication by others?

Partly

Reviewer Expertise:

The article is interesting. Minor changes are required. I believe that I have enough knowledge in this research field to assess the quality of its content.

I confirm that I have read this submission and believe that I have an appropriate level of expertise to confirm that it is of an acceptable scientific standard.

# Peer review: Reviewer response for version 1

**DOI:** 10.21956/openreseurope.15956.r32165

**Published:** 2023-07-12

**Authors:** Marina Krichevskaya

**Affiliations:** 1 Tallinn University of Technology, Tallinn, Estonia

A very relevant and interesting case study on whether the emission of nanoparticles from a photocatalytic air purifier is a problem.

Comments

 Abstract - “Photocatalytic air purifiers based on nano-titanium dioxide (TiO _2_) visible light activation provide a…” Just a note: titanium dioxide cannot be activated by visible light unless modified.

 Methods - The adhesion of particles depends on the coating method. Is at least general information about coating technology available? What are the characteristics (at least the size) of the TiO _2_N particles that were used for the coating?

Is the study design appropriate and does the work have academic merit?

Yes

Is the work clearly and accurately presented and does it cite the current literature?

Yes

If applicable, is the statistical analysis and its interpretation appropriate?

Not applicable

Are all the source data underlying the results available to ensure full reproducibility?

Yes

Are the conclusions drawn adequately supported by the results?

Yes

Are sufficient details of methods and analysis provided to allow replication by others?

Partly

Reviewer Expertise:

Photocatalysis, AOP

I confirm that I have read this submission and believe that I have an appropriate level of expertise to confirm that it is of an acceptable scientific standard.

# Peer review: Reviewer response for version 1

**DOI:** 10.21956/openreseurope.15956.r32161

**Published:** 2023-06-08

**Authors:** Mahshab Sheraz

**Affiliations:** 1 Advanced Nanomaterials & Textile Research Division, Korea Institute of Industrial Technology, Ansan-si, Gyeonggi-do, South Korea; 2 Hallym University, Chuncheon-si, Gangwon-do, South Korea

This study offers significant insights into the emission of nanoscale TiO _2_ particles from the Gearbox Wivactive air purifier. The conducted experimental setups and measurements significantly enhance our comprehension of particle emissions in the context of indoor air purification. According to the results, the air purifier exhibits minimal particle emission rates and does not present substantial exposure hazards within the evaluated parameters. Overall, the paper is well designed and organized.

 I would like to bring up a few points for consideration and propose a few improvements that would strengthen the study.

1. The introduction provides a good overview of photocatalytic oxidation for air purification. I would like to suggest including more contextual information on the current state of indoor air purification.
2. The study describes two distinct experimental configurations, Test 1 and Test 2, but it would be advantageous to provide a deeper explanation of the rationale behind each setup and the specific differences between them. In addition, the authors should explain why these particular configurations were selected and how they correspond to actual indoor conditions.
3. The sensitivity of the measurement instruments used in the study may have affected the ability to detect and quantify low particle concentrations accurately. This limitation should be addressed in future studies by employing more sensitive measurement techniques or instruments.
4. The authors mentioned that the release of TiO _2_ nanoparticles was not quantified due to low release rates below the instruments' LOQ. It is important to discuss the implications of this limitation and the potential impact on the study's conclusions. Suggestions for improving sensitivity or alternative measurement methods could be provided.
5. This study mainly focuses on the emission potential of nanosized TiO _2_ particles and their exposure under laboratory-simulated conditions. For future research, it is crucial to investigate the long-term exposure and effects in real-life indoor environments.

Overall, this study provides important insights into the release potential of nanosized TiO _2_ particles from a commercial indoor air purifier. The authors conducted the experiments diligently and presented the results clearly.

Is the study design appropriate and does the work have academic merit?

Yes

Is the work clearly and accurately presented and does it cite the current literature?

Yes

If applicable, is the statistical analysis and its interpretation appropriate?

Yes

Are all the source data underlying the results available to ensure full reproducibility?

Partly

Are the conclusions drawn adequately supported by the results?

Yes

Are sufficient details of methods and analysis provided to allow replication by others?

Partly

Reviewer Expertise:

Indoor air pollution control and managment, Air purification, Photocatalysts, Synthesis of Metal-organic framework (MOFs) materials for air purification, and other environmental remdiations.

I confirm that I have read this submission and believe that I have an appropriate level of expertise to confirm that it is of an acceptable scientific standard.

# Peer review: Reviewer response for version 1

**DOI:** 10.21956/openreseurope.15956.r29678

**Published:** 2022-07-29

**Authors:** Ridha Djellabi

**Affiliations:** 1 Universitat Rovira i Virgili, Tarragona, Spain

The authors have studied the release of N-doped TiO2 from the photocatalytic reactor for air purification under reasonable worst-case operational conditions. The release of photocatalytic nanoparticles (NPs) from coating surface is one of the capital issues raised against the wide application of photocatalytic technology because of the toxicity caused by such NPs. The current study reported that there was no release of TiO2 NPs from the air purifier “Gearbox Wivactive”.

 My comments are listed below:

- Does the authors synthesis N doped TiO2 at lab? It would be better to show the details of synthesis and characterization.
- The release rate of NPs is associated directly with the coating approach. Therefore, it would be nice to mention which kind of coating method was used, providing experimental details.
- In general, balancing between a good coating of NPs on surface and enhanced photoactivity is a challenge. More details regarding the photocatalytic efficiency of Gearbox Wivactive purifier could be added.

Is the study design appropriate and does the work have academic merit?

Yes

Is the work clearly and accurately presented and does it cite the current literature?

Yes

If applicable, is the statistical analysis and its interpretation appropriate?

Yes

Are all the source data underlying the results available to ensure full reproducibility?

Partly

Are the conclusions drawn adequately supported by the results?

Yes

Are sufficient details of methods and analysis provided to allow replication by others?

Partly

Reviewer Expertise:

Photocatalysis

I confirm that I have read this submission and believe that I have an appropriate level of expertise to confirm that it is of an acceptable scientific standard.
